# Supplementary material for: Deep Learning-Based Segmentation of Post-Mortem Human’s Olfactory Bulb Structures in X-ray Phase-Contrast Tomography
Source: Tomography. 2022 Jul 22;8(4):1854–68. doi: 10.3390/tomography8040156 (PMC9331385; doi:10.3390/tomography8040156)
Supplement: Supplementary file 1 [file tomography-08-00156-s001.zip › tomography-1731878-supplementary.pdf]

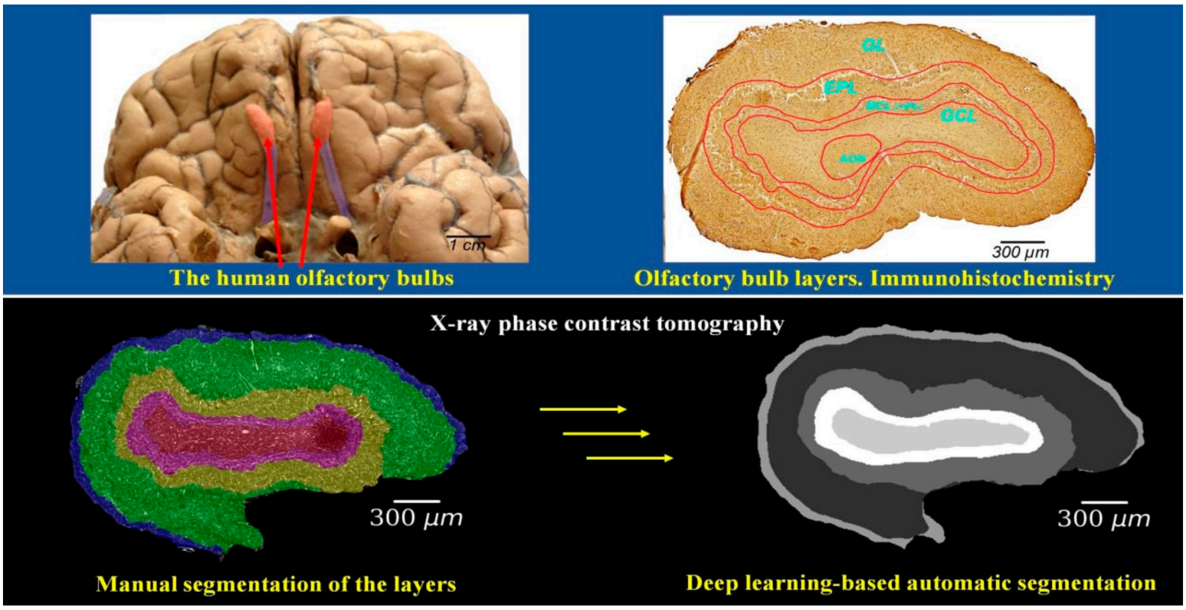
The human olfactory bulbs
1 cm
OL
EPL
GCL
300 μm
Olfactory bulb layers. Immunohistochemistry
X-ray phase contrast tomography
300 μm
Manual segmentation of the layers
300 μm
Deep learning-based automatic segmentation
